# Enhanced mixed proton and electron conductor at room temperature from chemically modified single-wall carbon nanotubes†

**DOI:** 10.1039/d2ra00521b

**Published:** 2022-03-18

**Authors:** Nurun Nahar Rabin, Md. Saidul Islam, Masahiro Fukuda, Junya Yagyu, Ryuta Tagawa, Yoshihiro Sekine, Shinya Hayami

**Affiliations:** Institute of Industrial Nanomaterials (IINa), Kumamoto University 2-39-1 Kurokami Chuo-ku Kumamoto 860-8555 Japan hayami@kumamoto-u.ac.jp; Department of Chemistry, Graduate School of Science and Technology, Kumamoto University 2-39-1 Kurokami Chuo-ku Kumamoto 860-8555 Japan; Priority Organization for Innovation and Excellence, Kumamoto University 2-39-1 Kurokami Chuo-ku Kumamoto 860-8555 Japan; International Research Center for Agricultural and Environmental Biology (IRCAEB) 2-39-1 Kurokami Chuo-ku Kumamoto 860-8555 Japan

## Abstract

Remarkably high mixed proton and electron conduction arising from oxidized single-wall carbon nanotubes at room temperature is demonstrated. The respective proton and electronic conductivities are 1.40 and 8.0 × 10^−2^ S cm^−1^ in the in-plane direction, and 3.1 × 10^−2^ and 1.1 × 10^−3^ S cm^−1^ in the out-of-plane direction, indicating their potential in a wide range of solid electrolyte membranes.

## Abstract 2

A chemically modified single-wall carbon nanotube showing efficient mixed proton and electron conduction at room temperature is demonstrated.

## Graphical abstract

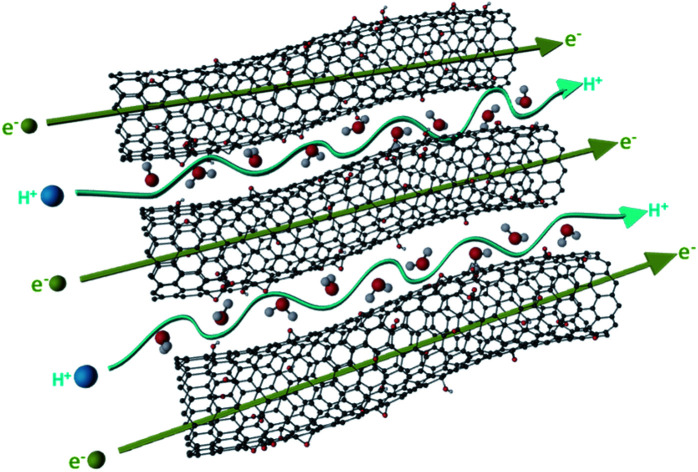

## Introduction

The design of solid electrolyte materials with controlled properties that include mixed ionic and electronic conduction properties is desirable for many potential applications ranging from energy storage to health technologies.^1,2^ In particular, mixed proton and electron-conducting (MPEC) materials can be used in a wide range of electrochemical devices such as hydrogen sensors and fuel cells, and in hydrogen separation, and electrolysis.^3–5^ During the past years, the external metal cations inserted in BaCeO_3_, LaYbO_3_, and La_6_WO_12_ type perovskite oxides are widely studied as MPEC while observing the high proton and electronic conductivity range as ∼10^−2^ to 10^−4^ and ∼10^2^ S cm^−1^, respectively.^6,7^ However the major barrier of these materials includes the high operating temperature (more than 650 °C) that largely limits their area of application. Besides, the chemical degradation of such materials takes place in the presence of water and carbon dioxide-containing air under the operating conditions. To achieve room temperature MPEC materials, some heterogeneous mixing of proton and electron conductors have been reported.^8–10^ Most of those MPEC materials are based on Nafion, which has obvious limitations in terms of very high cost. In addition, mechanical weakness and phase boundary-driven low performance are other drawbacks of such heterogeneous conductors. On the other hand, some single-phase mixed proton–electron conductor materials also reported that can overcome the phase boundary issue, however, the observed performance should be further improved to use such materials in practical application.^11–14^ Therefore, the development of efficient MPEC materials that can operate at room temperature is highly desirable for further advancement in the relevant research area.

During past decades, single-wall carbon nanotubes (SWCNT) are widely studied 1D carbon allotropes in different areas of application due to their unique physical and chemical properties.^15,16^ The SWCNTs are extremely mechanically stable and flexible. Hexagonal networks of CNTs consist of shells of carbon atoms that are sp^2^-hybridized. The hexagonal network is arranged helically within a tubular pattern. Because of the continuous π bonding networks in CNT, they are favorable for charge transfer. CNTs are good gas absorbers including H_2_, which makes them good candidates for membrane in fuel cells or some gas separator application. However, for making them more susceptible to utilize in desired applications, SWCNTs required chemical modification. The excellent electron conductivity of the SWCNT is well established. On the other hand, the oxygen functionalization in the carbon surface is associated with the transformation of sp^2^ to sp^3^ hybridization. As a consequence, the electronic conductivity of the materials decreases. However, the oxygen functionalization in the carbon materials is reported to show considerable proton conductivity under humidified conditions along with the adsorbed water layer associated with the oxygenated functional groups.^17,18^ Therefore, the incorporation of oxygen functional groups in the SWCNT surface is expected to show mixed conduction behaviour due to the oxygenated functional group in the surface being responsible for the proton conductivity and the carbon backbone in the CNT is responsible for the electron conductivity.

In some previous reports, the CNT and their derivatives have been utilized as an additive to prepare MPEC materials.^9^ However, to the best of our knowledge, the mixed proton–electron conductivity from the single-phase CNT-based materials is not reported to date. In this work, we have reported the excellent MPEC behaviors arising from the oxidized SWCNT materials. The pristine SWCNT shows very high electronic conductivity. After oxidizing SWCNT followed by freeze-drying (Ox-SWCNT-FD) results in a slight decrease in electronic conductivity while showing significant proton conductivity (excellent MPEC properties). Moreover, we have also compared the proton conductivity of associated oven-dried oxidized-SWCNT (Ox-SWCNT-OD).

## Experimental

The Ox-SWCNT was prepared from the pristine SWCNT using a modified hummer oxidation process.^21*a*^ Ox-SWCNT-FD and Ox-SWCNT-OD samples were prepared in the final stage of the drying process using freeze-drier and oven-drier, respectively. The details of the experimental processes are shown in ESI.† The prepared samples were characterized using XPS, SEM, TEM, and TGA analysis. The proton and electrical conductivity were measured using a pellet (30 MPa was applied to produce the pellet) of each sample. The proton conductivities (impedance) of the Ox-SWCNT-FD and Ox-SWCNT-OD were measured by the four-probe AC method using an impedance/gain phase analyzer (Solartron 1260) over the frequency range 1 to 10^6^ Hz. Bulk resistances were determined from the radius of the semicircle on the real axis. The electric conductivity was measured from the slope of current *versus* voltage curve (*I*–*V* curve) that represents the resistance in Ohm (Ω). The details of the MPEC measurement process are shown in ESI.†

## Results and discussion

**Fig. 1:**
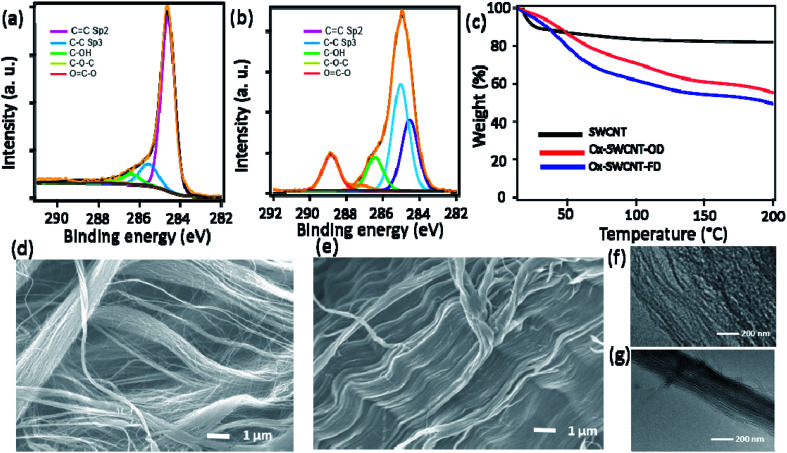
C 1s XPS spectra of (a) SWCNT and (b) Ox-SWCNT-FD. (c) TGA of SWCNT, Ox-SWCNT-OD and Ox-SWCNT-FD measured after keeping them 100% RH for 2 h. SEM images of (d) SWCNT and (e) Ox-SWCNT-FD. (f) TEM images of SWCNT and (g) Ox-SWCNT-FD.

**Fig. 2:**
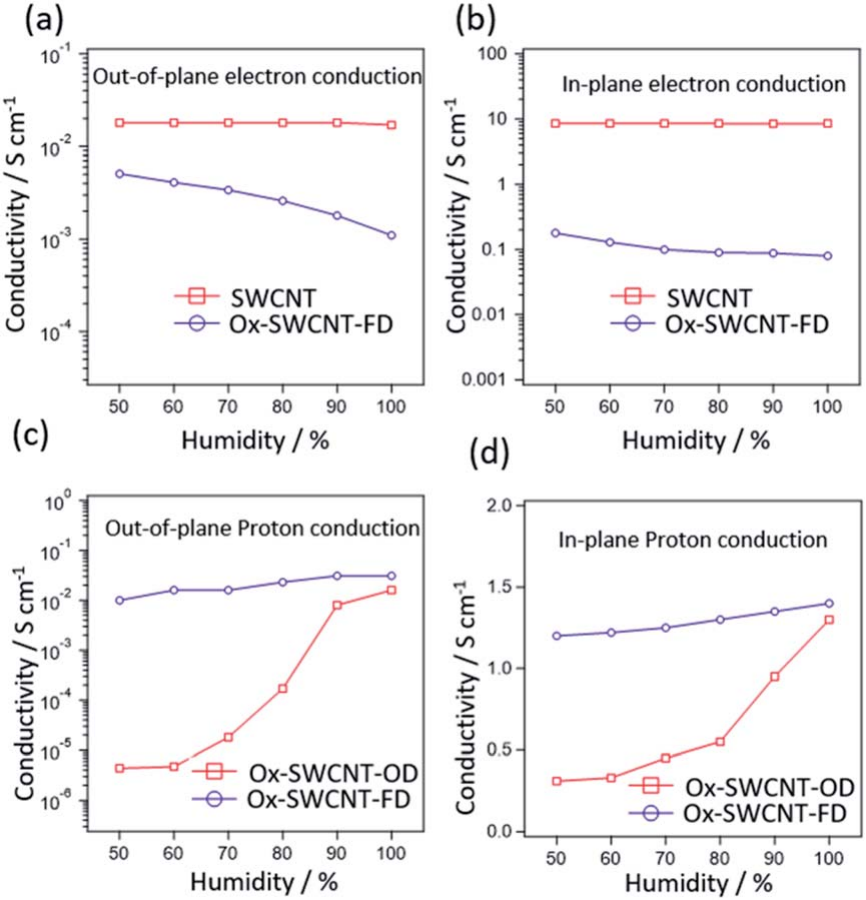
(a) Out-of-plane electronic conductivity of SWCNT and Ox-SWCNT-FD, (b) in-plane electronic conductivity of SWCNT and Ox-SWCNT-FD, (c) out-of-plane proton conductivity of Ox-SWCNT-FD and Ox-SWCNT-OD and (d) in-plane proton conductivity of Ox-SWCNT-FD and Ox-SWCNT-OD.

The development of oxygen functional groups in the surface of SWCNT was confirmed using XPS, FTIR spectra. Fig. 1a and b represent the C 1s XPS spectra of SWCNT and Ox-SWCNT-FD, respectively. Obviously, the peaks intensity for –COOH, -C-OH, and C–O–C functional groups at 288.8 eV, 286.4 eV, and 287.2 eV, respectively are largely increased in Ox-SWCNT-FD (Fig. 1b) which were insignificant in the C 1s XPS spectrum of pristine SWCNT (Fig. 1a).^18^ A slight shift in the peak position (–COOH peak at 288.5 in SWCNT *vs.* 288.8 in Ox-SWCNT-FD) might be attributed to the electron interaction by oxygen functionalization.^19,20^ Also, the estimated oxygen atomic percentage of 2.8% obtained from the SWCNT XPS survey spectra is increased to 36.14% after oxidation signifying the addition of oxygen-containing surface functional groups in the surface of SWCNT. The successful surface functionalization in Ox-SWCNT-FD can be also observed in FTIR spectra (Fig. S1†). The appearance of new peaks for Ox-SWCNT-FD at 2850–3000 cm^−1^, 1670–1750 cm^−1^, and 1050–1150 cm^−1^ can be assigned for the C–H groups, carbonyl (C=O), and C–O functional groups, respectively, whereas these peaks were absent/insignificant in the pure SWCNT. XPS and FTIR spectra of Ox-SWCNT-FD and Ox-SWCNT-OD show insignificant differences. The TGA, conducted after keeping all samples under 100% RH at room temperature for 2 h, of SWCNT and Ox-SWCNT are shown in Fig. 1c. The water uptake abilities of the SWCNT and OX-SWCNT-FD can be obtained through the weight loss up to 100 °C in TGA. Compared to the 16% weight loss for the SWCNT, the higher weight loss (37%) in Ox-SWCNT-FD confirms its higher water uptake ability which also indicates the presence of the oxygenated functional groups in Ox-SWCNT-FD responsible for uptake of a higher amount of water. The weight loss from SWCNT might be ascribed to the presence of inter-tube void space inside the SWCNT structure where the water molecules might be trapped during incubation at 100% RH for 2 h. The retention of the tubular structure of SWCNT after oxidation was confirmed through SEM and TEM images. Fig. 1d and e show the SEM images for SWCNT and Ox-SWCNT-FD, respectively. The tubes are more congested with each other after oxidation which might be attributed to the interaction among the polar surface functional groups developed from the oxidation process. Also, the corresponding EDX spectra in Fig. S2† support the XPS spectra for successful surface oxidation. The TEM images of SWCNT (Fig. 1f) and Ox-SWCNT-FD (Fig. 1g) further confirm the retention of the tubular structure after oxidation along with the nanotubes becoming more congested than that of pristine SWCNT. The above results suggest that the oxidation process is associated with the incorporation of oxygen-containing functional groups in SWCNT without decomposing its structure. The SWCNT and Ox-SWCNT-FD were used to measure the electronic and proton conductivity under 50–100% RH at room temperature. Fig. S3† shows the typical *I*–*V* curve for the estimation of electronic conductivity of SWCNT. The calculated value of electronic conductivity of pristine SWCNT and Ox-SWCNT-FD in the out-of-plane and in-plane directions are shown in Fig. 2 a and b, respectively. The out-of-plane electronic conductivity (Fig. 2a) of SWCNT is 1.8 × 10^−2^ S cm^−1^ at 50% RH while does not show a significant variation with increasing the RH. On the other hand, the electronic conductivity of Ox-SWCNT-FD is 5.1 × 10^−3^ S cm^−1^ at 50% RH, which gradually decreases with RH. At 100% RH, the conductivity value decreases to 1.1 × 10^−3^ S cm^−1^. The in-plane electronic conductivity (Fig. 2b) of both SWCNT and Ox-SWCNT-FD is about 2 orders higher than that of out-of-plane direction while showing similar behaviour under different RH conditions. The in-plane electronic conductivity of SWCNT is 8.7 S cm^−1^ at 50% RH which is almost stable at higher RH conditions. However, the in-plane conductivity of Ox-SWCNT-FD is 1.8 × 10^−1^ S cm^−1^ at 50% RH which decreases to 8.0 × 10^−2^ S cm^−1^ at 100% RH condition.

Fig. S4† shows the characteristic cole–cole plots (Nyquist plot) of impedance arising from Ox-SWCNT-FD at room temperature and 50–100% RH condition in the out-of-plane directions. The traces formed from plotting real (*Z*′) and imaginary parts (*Z*′′) of the impedance observe as distorted semi-circular curves followed by the existence of the second circle. This pattern indicates the typical proton conductive properties.^21*a*^ As expected, the SWCNT does not show any typical cole–cole plot of proton conduction implies their insignificant proton conduction under the experimental conditions employed. Proton conductivity has been measured for both Ox-SWCNT-FD and Ox-SWCNT-OD to observe the difference that arises due to the drying method. The calculated values of proton conductivity of Ox-SWCNT-FD and Ox-SWCNT-OD with respect to RH at room temperature in the out-of-plane direction and in-plane direction are shown in Fig. 2c and d, respectively. In all cases the proton conductivity value increase with increasing RH. This observation can be attributed to the enhanced amount of adsorbed water molecules responsible for the formation of hydrogen bonding for proton transportation. In the out-of-plane direction (Fig. 2c), the proton conductivity increases from 1.0 × 10^−2^ S cm^−1^ at 40% RH for Ox-SWCNT-FD to 3.1 × 10^−2^ S cm^−1^ at 100% RH condition. On the other hand, the proton conductivity values increase from 4.4 × 10^−6^ S cm^−1^ at RT and 40% RH to 1.6 × 10^−2^ S cm^−1^ at 100% RH. However, in the in-plane direction (Fig. 2d), Ox-SWCNT-FD shows exceptionally high proton conductivity of 1.2 S cm^−1^ at 40% RH which increases to 1.4 S cm^−1^ at 100% RH condition. On the other hand, Ox-SWCNT-OD shows the 3.1 × 10^−1^ S cm^−1^ at 40% RH to 1.3 S cm^−1^ at 100% RH. The above results suggest that the Ox-SWCNT-FD shows an exceptionally high proton and electronic conductivity.

Pristine SWCNT contains the tubular structure of sp^2^ hybridized carbon responsible for very high electron conductivity. Even though the surface oxidation of SWCNT results in a slight decrease in the electronic conductivity, nevertheless Ox-SWCNT-FD shows a significant value of electronic conductivity of 8.0 × 10^−2^ S cm^−1^ in the in-plane and 1.1 × 10^−3^ S cm^−1^ in the out-of-plane directions at room temperature and 100% RH. Compared with pristine CNT, the lower electrical conductivity of Ox-SWCNT-FD can be ascribed to the formation of oxygen functional groups on CNT surfaces. The reaction of oxygen with electron-donating carbon atoms located on the edge of the sp^2^ layers brings about a certain localization of the conduction electrons, which results in electrical insulation. However, a decrease in electronic conductivity of Ox-SWCNT-FD with increased RH is a consequence of water uptake. The sample swells after absorbing water, from which electrical connections within the carbon structure become disrupted.^13^

The development of materials with desired unique electrochemical properties is playing a key role to meet the current energy demand.^22–25^ Previously, our group has reported graphene oxide (GO)-based proton conductors for fuel cell application. Particularly, the oxygen-containing functional groups of GO are found to adsorb water in humidified conditions that can facilitate the proton conduction through the Grotthuss mechanism.^21^ Apart from the GO, some other chemically modified carbon materials including amorphous carbon and fullerene showing enhanced conductivity were reported.^17,18,26–28^ For example, Hatakeyama *et al.*, reported the liquid phase oxidation of coal while a significant amount of oxygenates sites was generated.^17^ The coal oxide showed well dispersion in the aqueous system. The surface carboxylic acid groups supported a proton conductivity of 3.9 × 10^−3^ S cm^−1^ at 90% RH in a comb-shaped microelectrode. The interface water trapped by the oxygenated functional group is attributed to the observed enhanced proton conductivity. Similarly, the oxygenated functional groups of Ox-SWCNT also adsorbed an adequate amount of water under humidified conditions. In fact, the higher water adsorption of the Ox-SWCNT is confirmed in TGA analysis (Fig. 1c). The observed high proton conductivity of Ox-SWCNT-FD can be attributed to the presence of an interconnected 1-D network with significant void space that promotes the maximum water uptake and hence shows the facile proton conduction track formation. Very recently, the freeze-dried route-driven 3D GO was found to show significantly higher proton conductivity compared to the traditional vacuum dried 2D GO.^29^ Herein, we also observed that the freeze-drying process of oxidized SWCNT can improve the proton conductivity over Ox-SWCNT-OD. The probable cause is explained in ESI.†

**Fig. 3:**
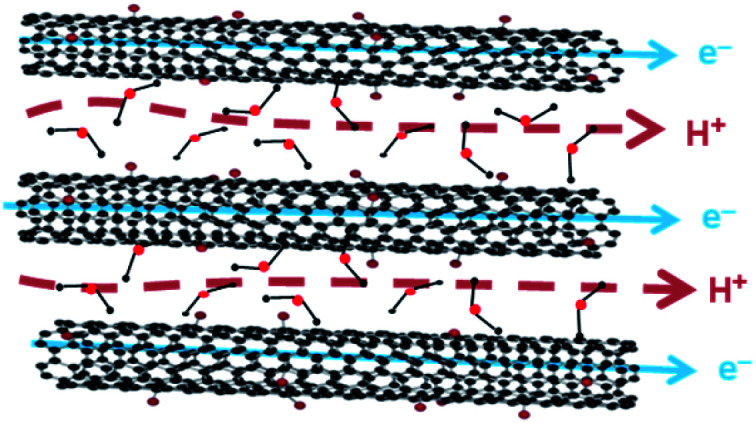
Proposed mixed proton and electron-conducting pathway of Ox-SWCNT-FD.

We believe that during the oxidation process only the part of the SWCNT surface undergoes in oxygenated functional groups with sp^3^ hybridized while the other part remains as non-oxidized with sp^2^ hybridized. For example, in the XPS survey spectra, the estimated oxygen content increased from 2.8% in SWCNT to 36.14% in Ox-SWCNT-FD. Therefore, the proton conduction is arising from the oxygenated functional groups of Ox-SWCNT-FD while the non-oxidized part of Ox-SWCNT-FD is responsible for the high electronic conductivity. As a result, the Ox-SWCNT-FD shows excellent MPEC properties. The proposed mixed proton and electronic conduction routes through Ox-SWCNT-FD are shown in Fig. 3.

Table 1 compares the competitive reported value for mixed proton and electron conductors operating at room temperature. Clearly, compared to the previously reported mixed conductors, the Ox-SWCNT-FD shows better performance which signifies their possible use in practical application in solid electrolyte membrane.

**Table 1:** Reported values for mixed proton and electron conductors

Materials	Proton conductivity (S cm^−1^)	Electronic conductivity (S cm^−1^)	Ref.
Nafion/((PDDA + Pt + GN)/PSS)	2.3 × 10^−3^	1.4 × 10^−2^	8
Nafion/aligned CNT	5.6 × 10^−4^	2 × 10^−3^	9
Nafion/rGO	2.2 × 10^−1^	2.20 × 10^−2^	10
rGO	2 × 10^−4^	2 × 10^−4^	11
Sulfonated-RGO (in-plane)	2 × 10^−2^	2 × 10^−2^	12
Oxidized porous carbon	3.15 × 10^−5^	1.68 × 10^−3^	13
Nickel(iii) dithiolene complex/1,4-naphthoquinone	7.2 × 10^−7^	4.1 × 10^−3^	14
Ox-SWCNT-FD (in-plane)	1.4	8.0 × 10^−2^	This work
Ox-SWCNT-FD (out-of-plane)	3.1 × 10^−2^	1.1 × 10^−3^	This work

## Conclusions

In summary, we successfully prepared a potential mixed proton–electron conductor from a single-phase Ox-SWCNT-FD that functions at room temperature. The material shows exceptionally high mixed proton and electron conductivity in both the in-plane and out-of-plane directions. The high-water uptake ability along with facile ions and charges conduction pathway that arises from the Ox-SWCNT can be attributed to the observed properties. We propose that the surface-functionalized SWCNT followed by a freeze-drying driven interconnected 1-D network with a facile ion/charge conduction pathway showing exceptionally high MPEC properties can be an ideal candidate for the next generation solid electrolyte materials for a wide application area.

## Conflicts of interest

There are no conflicts to declare.

## Supplementary Material

RA-012-D2RA00521B-s001
