# Supplementary material for: Enhanced mixed proton and electron conductor at room temperature from chemically modified single-wall carbon nanotubes
Source: RSC Adv. 2022 Mar 18;12(14):8632–6. doi: 10.1039/d2ra00521b (PMC8984934; doi:10.1039/d2ra00521b)
Supplement: RA-012-D2RA00521B-s001 [file RA-012-D2RA00521B-s001.pdf]

Electronic Supplementary Material (ESI) for RSC Advances.
This journal is © The Royal Society of Chemistry 2022

Enhanced mixed proton and electron conductor at room temperature from chemically modified single-wall carbon nanotubes

Nurun Nahar Rabin,[a] Md. Saidul Islam,[a,b] Masahiro Fukuda,[b] Junya Yagyu,[b] Ryuta Tagawa,[b] Yoshihiro Sekine, [b,c] and Shinya Hayami*[a, b, d]

[a] Institute of Industrial Nanomaterials (IINa), Kumamoto University, 2-39-1 Kurokami, Chuo-ku, Kumamoto 860-8555, (Japan).

[b] Department of Chemistry, Graduate School of Science and Technology, Kumamoto University, 2-39-1 Kurokami, Chuo-ku, Kumamoto 860-8555, (Japan).

[c] Priority Organization for Innovation and Excellence, Kumamoto University, 2-39-1 Kurokami, Chuo-ku, Kumamoto 860-8555 (Japan).

[d] International Research Center for Agricultural and Environmental Biology (IRCAEB)2-39-1 Kurokami, Chuo-ku, Kumamoto 860-8555, (Japan).

Synthesis of Ox-SWCNT: SWCNT (1g, 100-600μm in length, 3-5 nm in diameter, 800 m2/g surface area, ZEONANO SG101), $NaNO_3$ (fine meshed, 0.5 g) and H_2SO_4 (300 ml, 98%) was cooled to 0 °C by stirring in an ice bath for 1 h. 3.0 g finely meshed $KMnO_4$ powder was added slowly with vigorous stirring while keeping the temperature below 20 °C. After 30 min the mixture was warmed to 35±3 °C for 1.5 h. Then 100 ml water was added slowly, the temperature rose gradually and was kept in 95±3 °C for another 30 min. Then 600 ml water was added. Finally H_2O_2 (30%, 12 mL) was added to convert the unreacted permanganate and manganese dioxide into soluble sulfates. The mixture was centrifuged (3000 rpm, 10 min.) and the precipitate was washed with 5% HCl solution (1 time) and water (3 times).

Ox-SWCNT-OD and Ox-SWCNT-FD samples were obtained in the final stage of the drying process by using oven-drier (vacuum oven at 60 °C) and freeze drier, respectively. It can be noted that, in the current work, we mainly focused and highlighted the Ox-SWCNT-FD. However, Ox-SWCNT-OD is only briefly described to understand the superiority of the freeze-drying route over the traditional vacuum oven drying process in the ion conduction experiment.

Proton conductivity measurement: The proton conductivities (impedence) in both in-plane and out-of-plane direction of the Ox-SWCNT-FD and Ox-SWCNT-OD were measured by the four-probe AC method using an impedance/gain phase analyzer (Solartron 1260) over the frequency range 1 to 10^6 Hz. In a typical cell preparation for out-of-plane proton conductivity, both sides of each pellet were attached to a gold wire (50 μ m diameter, Tanaka Kikinzoku Kogyo) while covering the surface of each side with gold paste. Measurements were executed under controlled temperature and humidity using an incubator (SH-221, ESPEC). Bulk resistances were determined from the radius of the semicircle on the real axis. Proton conductivity (σ) was calculated according to the equation $\sigma = (1/R)*(d/A)$ where, R is the resistance (radius of the semicircular curve), d is the thickness of film sample and A is the area of the sample (gold paste coated part). For in-plane conductivity measurement, each pellet was placed on the top of a pair of metal electrodes. The conductivity was calculated using $\sigma = (1/R)*(d/T*L)$. Here, T is the thickness of pellet, d is the width (the distance between the electrodes), R is the measured resistance, and L is the length of the sample that is perpendicular to d.

Electric conductivity measurement: The electric conductivity was measured from the slope of current versus voltage curve (I-V curve) that represents the resistance in Ohm (Ω) while the slop is equal to 1/R. The in-plane and out-of-plane conductivity was measured using the same equation as proton conduction.

Comparison of Proton conductivity properties between the Ox-SWCNT-FD and Ox-SWCNT-OD: As we discussed in the experimental part that the only difference between the Ox-SWCNT-FD and Ox-SWCNT-OD is the drying method at the end of the oxidation process. Both the sample show identical XPS and FTIR indicates the surface functional groups does not alter due to the drying method. However, the TGA analysis indicates a little improvement in water uptake in the Ox-SWCNT-FD than that of Ox-SWCNT-OD (Figure 1c). In particular, during the freeze-drying process, the Ox-SWCNT suspension in aqueous media is frozen. On forming ice, the whole volume expands, and nearby particles are further separated and the relative positions of the particles become fixed and are no longer free to approach each other. This results in the interfacial tension between the water molecules and the particles in the solid being far weaker than that occurring between liquid water molecules and the particles. Therefore, the dried product might be associated with large void space resulting in improvement in the water uptake ability.[1]

The proton conductivity of Ox-SWCNT-FD and Ox-SWCNT-OD is comparable in the high humidity condition. However, at low humidity conditions, the Ox-SWCNT-FD has much higher proton conductivity than that of Ox-SWCNT-OD, which can be attributed to the facile proton conduction channel (resulting from the freeze-dried process) in Ox-SWCNT-FD that allow higher proton conduction even in relatively lower humidity. As in a recent report, we observed that the freeze-dried method can significantly improve the proton conductivity over the traditional vacuum oven drying process.[1] Therefore, herein we have also accomplished a similar study for oxidized SWCNT. The improved performance of Ox-SWCNT-FD than that of Ox-SWCNT-OD indicates that the freeze-drying method can be also effective to enhance the proton conductivity in other proton conductive systems.

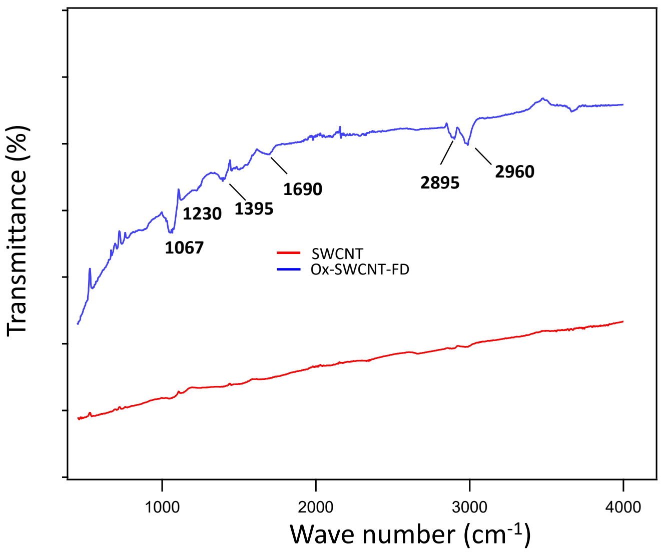

Figure S1: FT-IR spectra of SWCNT and Ox-SWCNT-FD

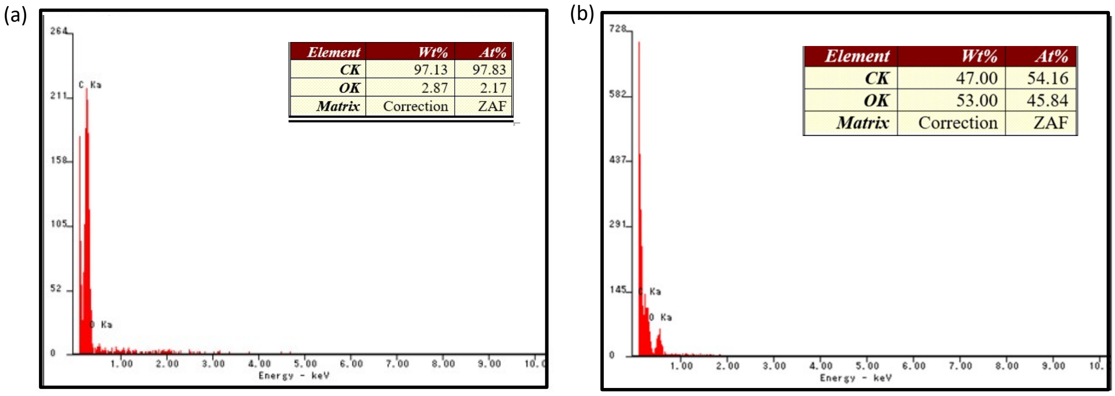

Figure S2: EDX spectra of a) SWCNT and b) Ox-SWCNT-FD

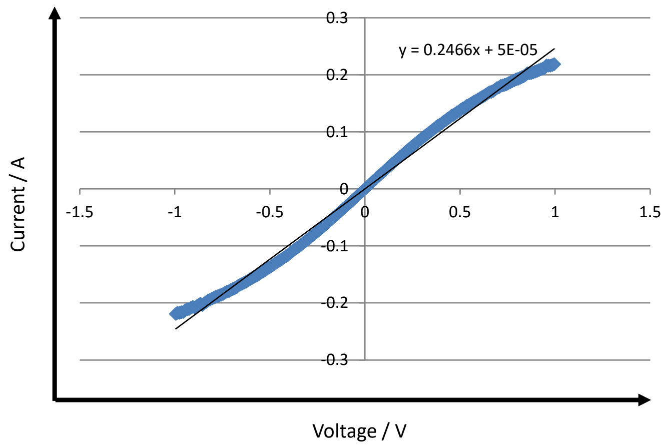

Figure S3: Representative I-V plot of pristine SWCNT measured in the in-plane direction at room temperature and 50% RH.

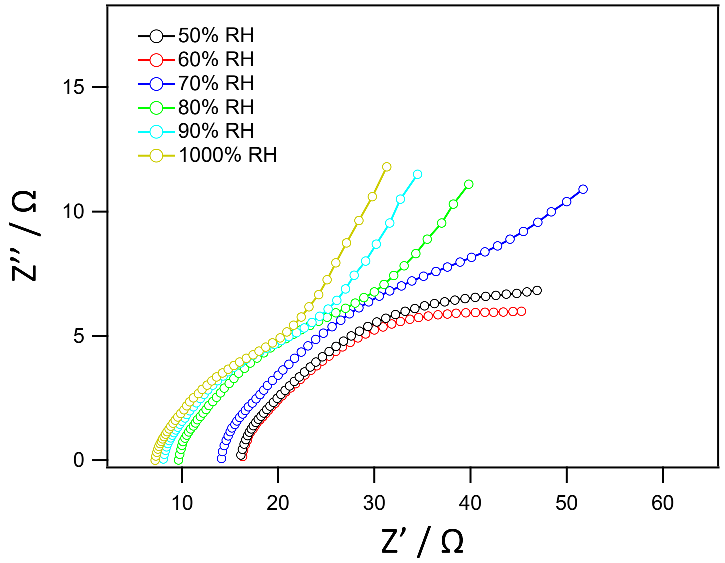

Figure S4: Representative cole-cole plot of pristine Ox-SWCNT-FD measured in the out-of-plane direction at room temperature and 50-100% RH.

Reference:

1. J. Yagyu, Md. S. Islam, Y. Shudo, M. Fukuda, H. Ushijima, J. Ohyama, S. Ida, L. F Lindoy and S. Hayami, ACS Appl. Energy Mater., 2021, 4, 6296.
